# Neuromuscular Responses of the Cranio-Cervico-Mandibular System During Professional “110th Targa Florio” Rally Competitions: An Electromyographic Study in Real-World Racing Conditions

**DOI:** 10.3390/dj14080474

**Published:** 2026-08-03

**Authors:** Davide Alessio Fontana, Salvatore Nigliaccio, Emanuele Di Vita, Antonio Scardina, Giuseppe Cutino, Giuseppe Geraci, Francesca Pusateri, Enzo Cumbo, Pietro Messina, Giuseppe Alessandro Scardina

**Affiliations:** Department of Precision Medicine in Medical, Surgical and Critical Care (e.PMre.C.C), University of Palermo, 90128 Palermo, Italy; davidealessiofontana@libero.it (D.A.F.); salvo.nigliaccio@gmail.com (S.N.); emanueledivita95@gmail.com (E.D.V.); antoscardi213@gmail.com (A.S.); cutinogiuseppe2002@gmail.com (G.C.); peppegeraci2015@libero.it (G.G.); francescapusateri24@gmail.com (F.P.); enzo.cumbo@unipa.it (E.C.); pietro.messina01@unipa.it (P.M.)

**Keywords:** motorsport, rally drivers, neuromuscular adaptation, surface electromyography, cranio-cervico-mandibular system, cervical stabilization, jaw clenching, motor control, athlete monitoring, sports performance

## Abstract

**Background/Objectives:** Professional motorsport imposes substantial biomechanical, postural, and cognitive demands that require continuous neuromuscular adaptations to ensure vehicle control and head stabilization during competition. Although the cranio-cervico-mandibular system may contribute to these adaptive responses, its role in rally performance remains poorly understood. This study aimed to investigate neuromuscular adaptations of the cranio-cervico-mandibular system in professional rally drivers under real-world racing conditions and to explore the relationship between masticatory muscle activity, cervical involvement, and jaw-clenching behavior. **Methods:** Twenty professional drivers and co-drivers participating in the 110th Targa Florio Rally were enrolled in the study. All participants underwent static surface electromyography (sEMG) assessment using the Teethan^®^ system to evaluate the masseter, anterior temporalis, and sternocleidomastoid muscles. During competition, dynamic masseter muscle activity was monitored using the dia-BRUXO^®^ system. Neuromuscular balance parameters, cervical involvement, masseter muscle activity, and clenching and grinding episodes were analyzed. **Results:** Participants showed a generally preserved baseline stomatognathic neuromuscular balance, with greater inter-individual variability in cervical involvement. During competition, clenching episodes were significantly more frequent than grinding episodes (*p* < 0.001). A moderate positive correlation was observed between Cervical Load and the number of clenching episodes (rho = 0.570, *p* = 0.011). No significant correlations were found between stomatognathic functional indices and dynamic bruxism-related parameters. **Conclusions:** Professional rally drivers exhibited increased masticatory muscle activity during competition, predominantly characterized by jaw clenching. The association observed between Cervical Load and clenching episodes suggests a statistical concomitance between cervical and masticatory muscle recruitment, which reaches high levels in these elite athletes but requires further longitudinal research to confirm a directional functional interaction in cranio-cervical stabilization during high-demand driving tasks.

## 1. Introduction

Professional motorsport is a sporting discipline characterized by substantial biomechanical, cognitive, and functional complexity. During competitive driving, drivers are simultaneously exposed to intense physical and psychological demands, including high attentional workload, rapid motor decision-making, mechanical vibrations, multidirectional accelerations, and continuous postural adjustments [1,2,3]. These conditions require highly efficient neuromuscular control to maintain head stability, cervical spine alignment, and overall body posture throughout performance.

In rally racing, unlike other motorsport disciplines conducted on more predictable circuits, drivers must continuously adapt to changing terrain conditions, sudden turns, jumps, vibrations, variations in grip, and lateral forces. These factors impose considerable biomechanical stress on the cranio-cervical region, which plays a fundamental role in visuospatial control, head orientation, and the accuracy of motor responses. Consequently, head stabilization represents a critical component of performance, as it enables drivers to maintain adequate visual and proprioceptive perception under high-intensity driving conditions [2,3,4].

In recent years, increasing attention has been directed toward the potential role of the stomatognathic system in neuromuscular regulation and postural control. Masticatory muscles, particularly the masseter and anterior temporalis muscles, are not exclusively involved in mastication but may also participate in broader motor strategies through functional interactions with the cervical region. From this perspective, the activation of masticatory muscles during high-demand sporting tasks should not be interpreted solely as a parafunctional phenomenon but also as a potential component of a neuromuscular stabilization strategy involving the cranio-cervico-mandibular complex [5,6,7,8,9,10,11,12,13,14,15,16]. Recent electromyographic investigations have demonstrated the usefulness of combining static and ambulatory EMG monitoring to characterize masticatory muscle activity and bruxism-related behaviors in clinical populations, supporting the role of objective neuromuscular assessment in the evaluation of stomatognathic function [17,18,19,20,21,22,23,24,25,26,27,28].

Awake jaw clenching has traditionally been classified within the spectrum of awake bruxism and associated with psychological factors such as stress, concentration, and cognitive hyperarousal [17,29,30,31,32,33,34,35,36]. However, in sports characterized by substantial motor demands, jaw clenching may assume a different functional significance. In particular, during rally driving, the need to stabilize the head despite vibrations, lateral accelerations, and rapid postural adjustments may promote co-activation between cervical and masticatory muscles, thereby contributing to neuromotor control of the cranio-cervical region [5,6,8,14].

Nevertheless, the transferability of these stabilizing effects remains highly debated within the broader scientific community, especially when transitioning from controlled laboratory settings to complex athletic tasks. While voluntary teeth clenching has consistently shown acute benefits in maximal isometric strength, early rate of force development (RFD), and static postural control under predictable perturbations [37,38,39], its role in dynamic stability remains highly debated.

Recent investigations in sport-specific domains have reported inconsistent or null effects of oral motor activity on dynamic balance control and coordination-heavy performance [40,41]. Consequently, in highly technical and high-speed environments, it remains unclear whether increased masseter co-contraction serves as an effective stabilization mechanism or rather represents an automatic, stress-induced neuromuscular reflex secondary to high cognitive load and physical pressure. Given these mixed findings, investigating real-world, extreme biomechanical tasks such as professional rally driving provides a unique exploratory opportunity to observe how the cranio-cervico-mandibular system behaves under concurrent mechanical and psychological stress.

Despite the growing interest in motorsport physiology, the specific behavior of the cranio-cervico-mandibular system during actual competitive driving has remained largely unexplored. The present study addresses this gap by investigating professional drivers and co-drivers under real-world racing conditions at the 110th Targa Florio Rally. Our central hypothesis is that the pronounced activation of masticatory muscles observed during competition represents a task-specific functional adjustment related to the driver’s stabilization strategies, which can be contextualized by evaluating its relationship with basal cranio-cervical neuromuscular features.

To test this hypothesis, the primary objective of this study was to quantitatively evaluate the surface electromyographic (sEMG) activity of the masticatory muscles during actual racing, clarifying whether this recruitment represents a transient, stress-induced parafunctional response or a functional adjustment.

As a secondary objective, the relationship between baseline cervical involvement, static masseter muscle activity, and dynamic jaw clenching behavior was examined to identify potential neuromuscular coordination patterns associated with competitive performance. To the best of our knowledge, this is the first study to simultaneously investigate static baseline and dynamic real-world electromyographic parameters of the cranio-cervico-mandibular system in professional rally drivers. 

## 2. Materials and Methods

### 2.1. Study Design

This exploratory observational study was conducted under real-world rally competition conditions. The protocol included a pre-race static electromyographic assessment of the cranio-cervico-mandibular system and dynamic electromyographic monitoring during competition. The methodological objective was to integrate a baseline functional assessment with an ecological recording of masseter muscle activity during competitive performance.

### 2.2. Participants

The study involved 20 professional and competitive rally drivers and co-drivers participating in the 110th edition of the Targa Florio Rally, a rally competition held in Sicily, Italy(14–16 May 2026). All participants had competitive motorsport experience and voluntarily agreed to participate after receiving detailed information regarding the study objectives and experimental procedures.

Eligible participants were adults officially registered for the competition, capable of completing the entire experimental protocol, and free from contraindications to the application of surface electrodes. Exclusion criteria included a history or diagnosis of temporomandibular disorders (TMDs), severe sleep or awake bruxism, the use of occlusal appliances during the competition, recent cranio-cervical trauma, technically incomplete or non-interpretable recordings, and significant electrode detachment during dynamic monitoring.

### 2.3. Ethical Considerations

The study was conducted in accordance with the principles of the Declaration of Helsinki. All participants received comprehensive information regarding the aims and procedures of the study and provided written informed consent prior to enrollment.

The study protocol was approved by the Territorial Ethics Committee of the Sicilian Region (Approval No. 197, 15 April 2026).

### 2.4. Static Surface Electromyography of the Cranio-Cervico-Mandibular System

Static electromyographic assessment of the cranio-cervico-mandibular system was performed using a six-channel surface electromyography (sEMG) device (Teethan^®^, Teethan S.p.A., Milan, Italy). Examinations were conducted during the pre-race phase in a controlled environment according to a standardized protocol [17,18,19,20,21,22,23,24,25].

Disposable surface electrodes were bilaterally positioned over the masseter, anterior temporalis, and sternocleidomastoid muscles. Prior to electrode placement, the skin was cleaned with an alcohol solution to reduce skin impedance and improve signal quality. Electrode placement was performed by the same clinical team to minimize inter-operator variability.

The recording protocol included maximum voluntary clenching on cotton rolls, clenching in habitual intercuspation, an occluso-cervical assessment, and a chewing test using chewing gum. During data acquisition, participants remained seated in a standardized position with a natural head posture and forward gaze.

Electromyographic indices related to neuromuscular symmetry, occlusal load distribution, cervical involvement, and masticatory functional coordination were analyzed, including *Temporalis Percentage Overlapping Coefficient* (temporalis POC), *Masseter Percentage Overlapping Coefficient* (masseter POC), *Barycenter of Force* (BAR), *Torsional Index* (TORS), IMPACT, *Asymmetry Index* (ASIM), *Global index of neuromuscular balance* (GNE), *Sternocleidomastoid Percentage Overlapping Coefficient* (sternocleidomastoid POC), Cervical Load, and *Symmetrical Mastication Index* (SMI) [18,21,24].

### 2.5. Dynamic Electromyographic Monitoring During Competition

Dynamic monitoring of masseter muscle activity during competition was performed using a portable EMG-Holter device (dia-BRUXO^®^, Bios Research Center, Milan, Italy). The device was applied before the competitive session and maintained throughout the race to record masseter muscle activity under real-world racing conditions [17,42,43].

For each participant, activity of the left masseter muscle was recorded using a disposable bipolar surface electrode. The selection of the left masseter was determined by the technical characteristics of the device and the need to standardize electrode placement across participants. Signal quality was verified using the dedicated software before monitoring commenced.

At the end of the competition, data were downloaded and analyzed using the device-specific software. Quantitative indices of masseter muscle activity and clenching behavior were considered, including the Masseter Time Index (MTI), Masseter Work Index (MWI), Masseter Personal Index (MPI), Bruxism Time Index (BTI), Bruxism Work Index (BWI), Bruxism Personal Index (BPI), number of clenching episodes, and number of grinding episodes. The dynamic parameters provided by the Dia-BRUXO device are quantitative monitoring variables and currently lack universally accepted normative reference values. Therefore, they were interpreted descriptively and in relation to the other electromyographic measures collected in the present study.

### 2.6. Methodological Considerations Regarding the Devices

The combined use of static electromyography and dynamic monitoring allowed for an integrated assessment of drivers’ neuromuscular behavior both at baseline and under real competitive conditions. However, both methodologies present certain limitations.

The Dia-BRUXO^®^ system records activity from a single masseter muscle and does not allow simultaneous bilateral monitoring or direct recording of temporalis and cervical muscle activity during competition. Consequently, it does not permit direct demonstration of cranio-cervico-mandibular co-activation during racing. Furthermore, dynamic recordings acquired in rally environments may be influenced by vehicle vibrations, body movements, perspiration, and motion artifacts.

Surface electromyographic recordings are also sensitive to technical variables such as skin impedance, subcutaneous tissue thickness, electrode positioning, and electrode-skin contact quality [20,21,22,23,24]. To minimize these influences, standardized procedures for skin preparation, electrode placement, and signal verification were adopted. To minimize the risk of measurement errors or technical artifacts due to vehicle vibrations and sudden body movements, the Dia-BRUXO^®^ was physically stabilized by the compressive, tight fit of the fireproof balaclava and the full-face racing helmet. Furthermore, the proprietary software algorithm distinguished true jaw-clenching and parafunctional episodes from baseline background noise or instantaneous mechanical spikes by applying specific physiological thresholds based on signal amplitude and continuous duration.

### 2.7. Experimental Protocol

Each participant underwent a standardized assessment protocol conducted by the same clinical team to minimize inter-operator variability. During recruitment, a complete medical and dental history was collected. Subsequently, static surface electromyographic recordings of the cranio-cervico-mandibular system were performed before competition.

After skin cleansing with an alcohol solution, surface sensors were applied by a specialized clinical team according to standardized anatomical landmarks: over the anterior portion of the temporalis muscle, over the belly of the superficial masseter midway between the zygomatic arch and the gonial angle, and over the upper third of the sternocleidomastoid muscle belly. In all cases, bipolar electrodes were oriented parallel to the muscle fibers.

Static recordings were acquired under standardized conditions, including maximum voluntary clenching on cotton rolls, clenching in habitual intercuspation, occluso-cervical assessment through head rotation, and a chewing test using chewing gum. Cotton-roll recordings were used as a reference condition to minimize occlusal influences and assess baseline neuromuscular symmetry.

During static EMG acquisition, participants were instructed to maintain a standardized seated posture with a natural head position and forward gaze. For cervical assessment, participants performed right and left head rotations. No external force-feedback system was used to standardize maximal voluntary contraction intensity; therefore, contractions were guided through verbal instructions and real-time signal monitoring.

On the day of competition, during both the shakedown and race stages, the dia-BRUXO^®^ EMG-Holter device (Biotech Novations S.r.l., Sanremo, Imperia, Italy) was applied. Participants were instructed to wear the device continuously throughout the competitive session and to remove it only if strictly necessary. Upon completion of recording, the device was returned for data extraction and analysis.

### 2.8. Limitations

Finally, due to sample size constraints and to preserve adequate statistical power, drivers and co-drivers were evaluated within the same analytical group. Although both crew members share a highly comparable profile of physical and psychological stress within the cockpit, future larger-scale investigations should consider role-specific stratified analyses to identify potential subtle differences in neuromuscular recruitment patterns.

### 2.9. Statistical Analysis

All data obtained from static and dynamic electromyographic recordings were entered into a dedicated spreadsheet (Microsoft Excel 2021, Microsoft Corp., Redmond, WA, USA) and subsequently analyzed using XLSTAT software (version 2025.1; Addinsoft, New York, NY, USA).

Descriptive statistics were calculated for all variables, including mean, standard deviation, median, interquartile range, minimum, and maximum values.

Data distribution was assessed through graphical inspection and the Shapiro–Wilk normality test.

Given the limited sample size and the non-normal distribution of several electromyographic variables, non-parametric statistical tests were used for inferential analyses.

The comparison between clenching and grinding episodes was performed using the Wilcoxon signed-rank test.

Associations between static and dynamic parameters were assessed using Spearman’s rank correlation coefficient. Specifically, relationships between Cervical Load and clenching episodes, Cervical Load and A-MTI, SMI and A-BPI, and GNE and A-BPI were investigated.

Dynamic parameters obtained through the dia-BRUXO^®^ system were used to quantify masseter muscle activity as well as clenching and grinding episodes during competition (Table 1). Static electromyographic parameters obtained through the Teethan^®^ system were analyzed descriptively and compared with physiological reference values provided by the manufacturer. It is worth noting that the parameters used are already widely cited in the literature and considered reliable and reproducible [44,45] (Table 2 and Table 3).

Given the exploratory nature of the study and the limited sample size, no formal correction for multiple comparisons was applied. Therefore, inferential findings should be interpreted as hypothesis-generating rather than as definitive evidence of causal relationships.

## 3. Results

### 3.1. Descriptive and Static Electromyographic Analysis

Pre-race static electromyographic assessment revealed a substantial preservation of baseline stomatognathic neuromuscular balance in most participants, despite marked inter-individual variability.

During the static occlusal assessment, mean values for the temporalis Percentage Overlapping Coefficient (POC) and masseter POC were 84.6 ± 4.17 and 83.4 ± 7.01, respectively. The global neuromuscular equilibrium index (GNE) showed a mean value of 85.9 ± 3.18. The Wilcoxon signed-rank test revealed a slight but statistically significant reduction in temporalis POC compared with physiological reference values (*p* = 0.044), whereas masseter POC did not differ significantly from physiological reference values (*p* = 0.091).

The BAR and TORS indices showed no statistically significant deviations from reference values, suggesting an overall preservation of occlusal load distribution and torsional control under baseline conditions.

Analysis of the cervical region revealed a mean sternocleidomastoid POC value of 78.3 ± 10.1 and a mean Cervical Load value of 17.5 ± 16.4. These findings demonstrated greater inter-individual variability in cervical involvement compared with static occlusal parameters (Table 4).

### 3.2. Masticatory Parameters

During the chewing test, chewing frequency was largely preserved during both right- and left-sided mastication, with mean values of 1.61 ± 0.163 Hz and 1.72 ± 0.192 Hz, respectively.

The SMI showed a mean value of 51.4 ± 18.7, indicating a relatively preserved functional masticatory coordination. In contrast, the IMPACT indices related to the temporalis and masseter muscles exhibited considerable inter-individual variability (Table 5).

### 3.3. Dynamic Monitoring During Competition

Dynamic electromyographic monitoring using the dia-BRUXO^®^ system revealed an increase in masseter muscle activity during rally competition. Mean recorded values were 19.4 ± 9.83 for the A-MTI, 14.6 ± 7.43 for the A-MPI, and 6.57 ± 18.5 for the A-BPI.

The mean number of clenching episodes was 37.9 ± 46.2, whereas the mean number of grinding episodes was considerably lower, at 6.53 ± 12.1.

The Shapiro–Wilk test indicated a non-normal distribution for all dynamic parameters recorded by the dia-BRUXO^®^ system, highlighting substantial inter-individual variability in the patterns of masseter muscle activation observed during competition (Table 6).

**Figure 1:**
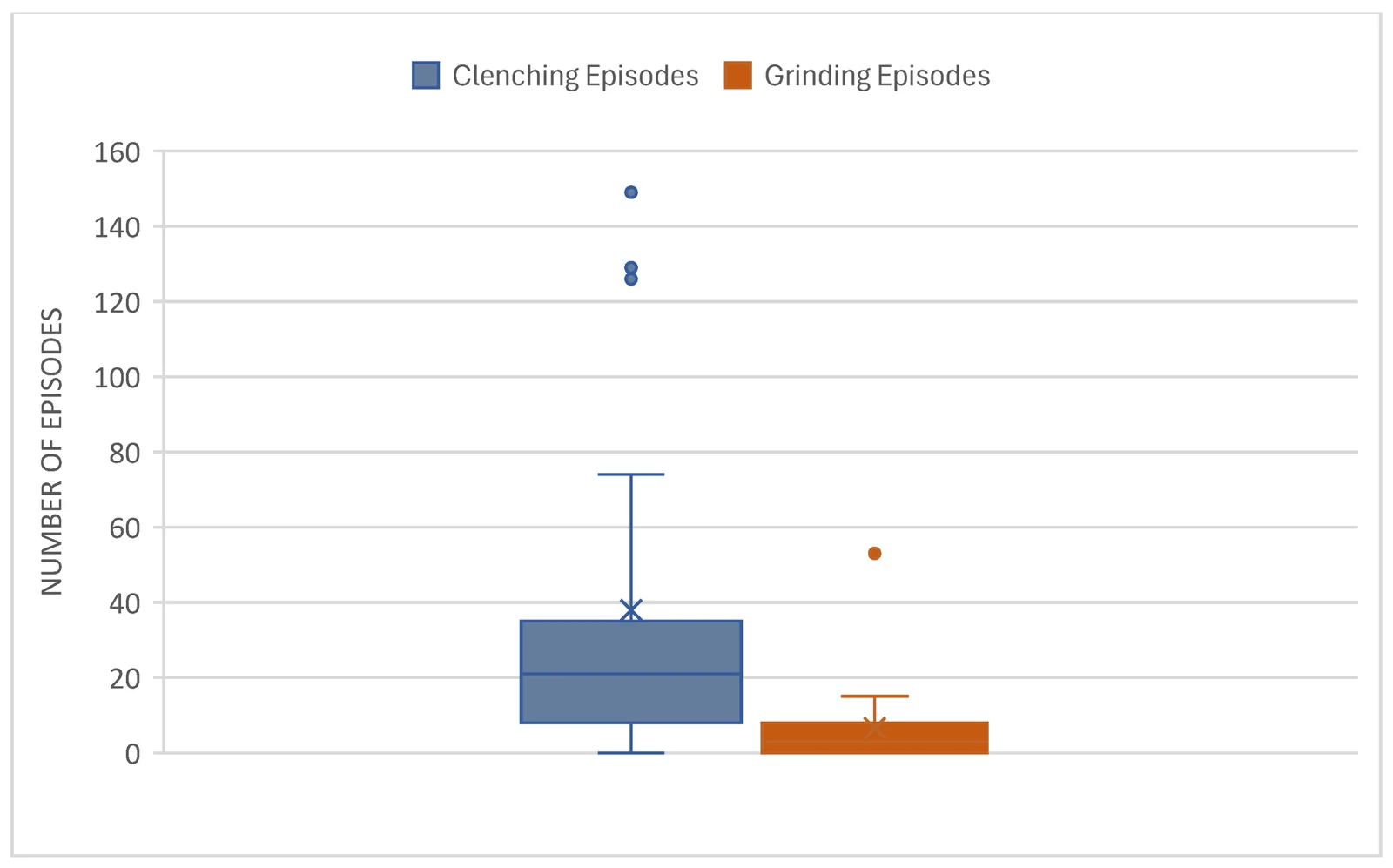
Comparison of Clenching and Grinding Episodes During Rally Competition. Boxplot showing the number of clenching and grinding episodes recorded during the competition. The number of clenching episodes was significantly higher than the number of grinding episodes (Wilcoxon signed-rank test, *p* < 0.001).

The Wilcoxon signed-rank test revealed a significantly higher number of clenching episodes compared with grinding episodes (W = 152; *p* < 0.001), indicating that masseter muscle activity during competition was predominantly characterized by sustained jaw-clenching behavior rather than grinding activity (Figure 1).

These findings indicate that the masseter muscle activity observed during competition was predominantly characterized by static jaw-clenching episodes rather than dynamic grinding behavior.

### 3.4. Relationship Between Cervical Involvement and Jaw Clenching

**Figure 2:**
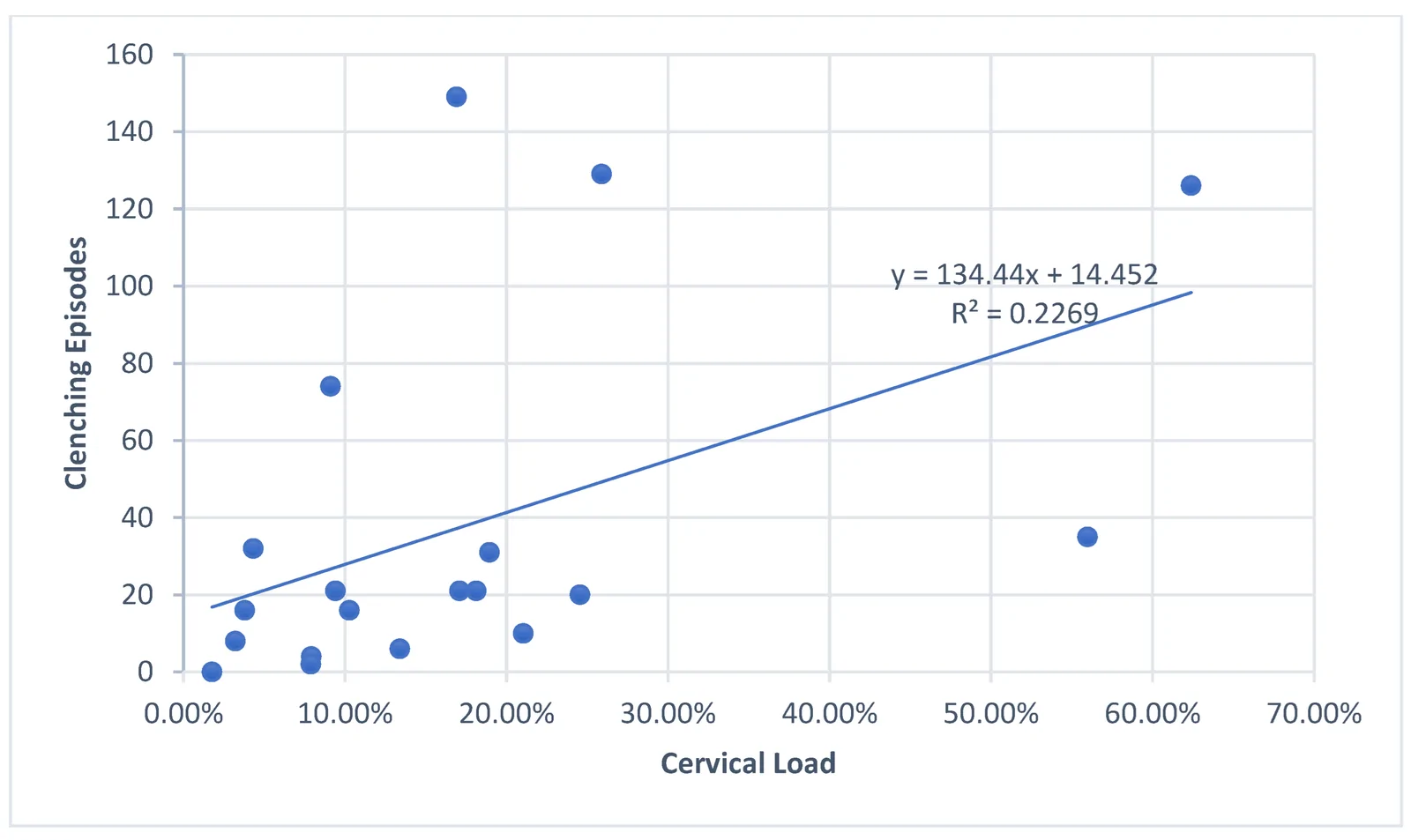
Correlation Between Cervical Load and Clenching Episodes in Professional Rally Drivers. Scatter plot showing the positive correlation between Cervical Load and the number of clenching episodes recorded during the competition (Spearman’s rho = 0.570; *p* = 0.011).

Spearman’s correlation analysis revealed a moderate and statistically significant positive correlation between Cervical Load and the number of clenching episodes (rho = 0.570; CI: 0.124 to 0.822; *p* = 0.011) (Figure 2).

At the same time, the correlation between Cervical Load and A-MTI showed only a positive trend that did not reach statistical significance (rho = 0.304; *p* = 0.206). Furthermore, no significant correlations were observed between SMI and A-BPI (rho = −0.014; *p* = 0.954) or between GNE and A-BPI (rho = 0.020; *p* = 0.934) (Table 7).

## 4. Discussion

The present study investigated cranio-cervico-mandibular neuromuscular activity in professional rally drivers under real-world competitive conditions by integrating a pre-race static electromyographic assessment with dynamic monitoring of masseter muscle activity during performance. The main findings indicate that drivers develop a substantial activation of the masticatory musculature during competition, predominantly characterized by static jaw-clenching episodes, and that this activity could be associated with cervical involvement.

The most relevant finding of the study is the positive correlation observed between Cervical Load and the number of clenching episodes. This association suggests that participants exhibiting greater cervical muscle recruitment during the standardized MVC assessment also experienced a higher number of jaw-clenching episodes during competition. From a functional perspective, this pattern may reflect an integrated cranio-cervico-mandibular co-activation strategy aimed at head control and neuromotor stabilization under conditions of high biomechanical demand [5,6,7,8,9,10,11,12,13,14]. Although this association reached statistical significance, it should be interpreted cautiously because no adjustment for multiple comparisons was applied, consistent with the exploratory nature of the study; therefore, confirmation in larger, adequately powered investigations will be necessary. Finally, it is important to underscore that the observed association does not imply a direct causal relationship.

Although the observed correlation supports the hypothesis of a functional relationship between cervical involvement and masseter muscle activity, the observational design of the present study does not allow causal inferences to be drawn. Therefore, the hypothesis of cranio-cervico-mandibular co-activation as an adaptive mechanism should be considered preliminary and requires further experimental confirmation.

In professional motorsport, head stabilization represents a fundamental component of performance [1,2,3,4,14,46,47,48]. Drivers must maintain highly precise visual and postural control while being exposed to vibrations, lateral accelerations, rapid changes in direction, and conditions of heightened attentional demand. Within this context, jaw clenching may not represent solely a parafunctional behavior but could also constitute a task-specific response embedded within a broader neuromuscular stabilization strategy [5,6,14,17,33].

It is worth noting that the severe mechanical vibrations and intense vehicle jarring characteristic of rally competitions, especially on non-circuit open roads, represent a unique environmental stressor. From a physiological standpoint, these extreme conditions do not merely act as external noise, but actively trigger genuine, subconscious neuromuscular bracing. The electromyographic activity recorded during the race reflects a complex, multifactorial response. While it may represent the stomatognathic system adapting to stabilize the head and jaw against continuous mechanical impacts, it is equally crucial to consider the significant impact of psychological factors. High cognitive workload, intense concentration, and emotional stress are well-documented triggers for awake bruxism and sustained masseter muscle contraction. Under real-world competitive conditions, the massive psycho-physical workload and heightened attentional demand could independently drive, or heavily amplify, these jaw-clenching episodes. Therefore, the observed muscular activation likely represents an intertwined response where biomechanical bracing and cognitive-stress-induced neuromuscular activation coexist, a dual nature that warrants further investigation using simultaneous psychophysiological parameters (e.g., heart rate variability or cortisol levels). The significantly higher prevalence of clenching episodes compared with grinding episodes confirms that the activity observed during competition was not primarily characterized by dynamic mandibular movements but rather by sustained or prolonged contractions of the masseter muscles. This finding is particularly noteworthy because, unlike grinding, jaw clenching has been hypothesized to theoretically contribute to increased functional stiffness of the cranio-cervico-mandibular complex. However, whether this mechanism directly enhances head stability during tasks requiring high levels of postural control remains strictly hypothetical, as dynamic cervical movement and stability were not objectively measured in our protocol.

The predominance of jaw-clenching episodes observed in the present study is consistent with previous electromyographic investigations reporting increased awake masticatory muscle activity in conditions characterized by sustained neuromuscular activation [17,33,42].

Another relevant finding is the relative preservation of static occlusal parameters and functional masticatory coordination. The values of masseter POC, GNE, and SMI suggest that the increase in masseter muscle activity during competition was not necessarily associated with impairment of baseline stomatognathic neuromuscular balance [17,18,19,20,21,22,23,24,25,26,27,28]. Furthermore, the absence of significant correlations between the baseline electromyographic indices (SMI, GNE, POC) and the dynamic racing parameters warrants a deeper physiological interpretation. While it could superficially appear to weaken the hypothesis of a unified cranio-cervico-mandibular co-activation, this lack of association likely underscores the functional resilience of the stomatognathic system. It demonstrates that the substantial increase in masseter muscle activity during competition does not chronically destabilize or alter the pilot’s baseline neuromuscular balance. Instead, this dissociation suggests that jaw clenching acts as a highly localized, task-specific, and transient response to acute biomechanical and cognitive stressors. The compensatory mechanisms of the masticatory apparatus appear efficient enough to absorb the intense workload of the race without carrying over functional imbalances into static occlusal conditions, reinforcing the adaptive nature of this neuromuscular behavior. The substantial inter-individual variability observed across the dynamic electromyographic parameters suggests that participants may adopt different neuromuscular responses during competition. Such variability may reflect differences in competitive experience, individual motor control characteristics, psychophysiological status, competitive stress, vehicle characteristics, or specific race conditions. From a clinical and sports medicine perspective, this heterogeneity represents an important finding, as it may facilitate the future identification of individual profiles of neuromuscular adaptation or overload. Because the primary analyses were based on Spearman’s rank correlation, the influence of extreme observations was mitigated. Nevertheless, given the limited sample size, the stability of these associations should be confirmed in larger cohorts, where sensitivity analyses may further assess the impact of individual observations.

From a sports medicine perspective, the findings of the present study provide new insights into the neuromuscular monitoring of athletes involved in motorsport. An integrated assessment of the cranio-cervico-mandibular system may contribute to the identification of cervical overload patterns, compensatory strategies, and potential risk factors for muscular fatigue or functional disorders [49,50]. Furthermore, monitoring masseter muscle activity during performance may represent an additional tool for understanding individual neuromuscular responses to competitive demands.

The present study has several limitations that warrant caution. First, the observational, cross-sectional design prevents the evaluation of long-term adaptations, while the relatively small sample size and the absence of a control group limit the immediate generalizability of the findings. In addition, objective measures of driving performance, vehicle biomechanical load, accelerations, vibrations, and psychophysiological stress were not collected, which would have allowed a more precise interpretation of the relationship between muscle activation and competitive demands. Dynamic monitoring during competition was limited to a single masseter muscle, without simultaneous recording of cervical muscle activity, thereby limiting the possibility of directly demonstrating cranio-cervico-mandibular co-activation during racing. Finally, surface electromyographic recordings acquired under real racing conditions may be affected by motion artifacts, vehicle vibrations, and technical variables related to electrode placement [18,19,20,21,22,23,24,25,26,27,28].

Despite these limitations, the study demonstrates high ecological validity, as recordings were obtained during an official competition and under authentic performance conditions. This represents a significant strength, considering the practical challenges associated with collecting neuromuscular data from professional athletes during real competitive motorsport events.

Future studies should include larger sample sizes, control groups, bilateral multichannel EMG monitoring during competition, simultaneous recording of cervical muscles, kinematic analyses of head and cervical spine movements, vehicle biomechanical data, stress biomarkers, and heart rate variability measures. Such approaches would provide a more comprehensive understanding of the role of cranio-cervico-mandibular co-activation in motorsport performance and help distinguish functional adaptation from potential neuromuscular overload.

## 5. Conclusions

The present study suggests that professional rally drivers develop a cranio-cervico-mandibular neuromuscular response under real competitive conditions, characterized by increased masseter muscle activity and a predominance of static jaw-clenching episodes over grinding behavior.

The positive correlation observed between Cervical Load and clenching episodes supports the hypothesis that activation of the masticatory muscles may be associated with cervical involvement during competitive driving. Within this specific sporting context, jaw clenching may therefore represent not only a parafunctional phenomenon but also a component of an adaptive neuromuscular strategy hypothesized to contribute to cranio-cervical stabilization.

Despite the increase in masseter muscle activity during competition, static occlusal parameters and functional masticatory coordination remained relatively preserved, suggesting the presence of compensatory mechanisms capable of maintaining stomatognathic system efficiency even under conditions of substantial neuromotor demand.

Overall, these findings contribute to a broader understanding of the role of the cranio-cervico-mandibular system in the physiology of motorsport performance and open new avenues for research in sports medicine, neuromuscular monitoring, and functional optimization of professional athletes.

Further studies involving larger cohorts, multichannel electromyographic monitoring during competition, and the integration of biomechanical and psychophysiological parameters will be necessary to confirm the role of cranio-cervico-mandibular co-activation as a stabilization strategy during professional motorsport competitions.

The cranio-cervico-mandibular system may represent a previously underappreciated component of the neuromuscular adaptation processes involved in motorsport performance.

## Figures and Tables

**Table 1:** Dia-Bruxo Parameters.

*Index*	*Full Name*	*Definition*	*Unit*	*Represents*
***MTI***	Masseter Time Index	Percentage of time showing masseter muscle activity with respect to the total EMG-Holter recording duration.	%	Temporal extent of overall masseter activity.
***MWI***	Masseter Work Index	Percentage of muscular work generated by the masseter muscle relative to the potential work that would have been produced if the maximum recorded power peak had been continuously maintained for the entire EMG-Holter monitoring period.	%	Overall muscular workload or functional performance of the masseter.
***MPI***	Masseter Personal Index	Integrated electromyographic index expressing the subject-specific activation profile of the masseter muscle during prolonged dynamic recording, derived from cumulative muscular activity over time.	%	Individual neuromuscular recruitment and adaptive masseteric workload during competitive activity.
***BTI***	Bruxism Time Index	Percentage of time characterized by bruxism episodes during the entire EMG-Holter monitoring period.	%	Temporal extent of bruxism activity.
***BWI***	Bruxism Work Index	Percentage of muscular work performed during bruxism episodes relative to the potential work that would have been produced if the highest recorded power peak had been continuously maintained throughout all bruxism episodes.	%	Muscular effort or intensity of bruxism.
***BPI***	Bruxism Personal Index	Composite indicator derived from BTI and BWI, representing the individual’s overall level of bruxism, integrating both time and intensity parameters.	Dimensionless	Global bruxism severity

**Table 2:** Teethan Parameters (Occluso-Cervical Test).

*Index*	*Full Name*	*Definition*	*Unit*	*Represents*
***POC TA***	Percentage Overlapping Coefficient—Temporalis Anterior	Quantifies the bilateral symmetry of the anterior temporalis muscles during clenching, expressed as a percentage of overlap between left and right EMG activity.	%	Neuromuscular coordination and functional symmetry of the temporalis muscles.
***POC MM***	Percentage Overlapping Coefficient—Masseter Muscles	Quantifies the bilateral symmetry of the masseter muscles during clenching, expressed as a percentage of overlap between left and right EMG activity.	%	Neuromuscular coordination and balance of the masseter muscles.
***BAR***	Barycenter of Force	Indicates the occlusal force center of gravity, calculated from the relative activation of right and left masseter and temporalis muscles.	%	Distribution of occlusal load between dental arches.
***IMPACT***	Impact Index	Represents the overall magnitude of muscular activity during clenching, derived from the sum of EMG amplitudes relative to a normative reference.	%	Global muscle activation level/functional tone.
***TORS***	Torsional Index	Expresses the degree of torsional balance between contralateral pairs of muscles (right temporalis + left masseter vs. left temporalis + right masseter).	%	Bilateral torsional control and occlusal coordination.
***ASIM***	Asymmetry Index	Measures the lateral deviation of total EMG activity between right and left muscle groups.	%	Direction and magnitude of functional asymmetry.
***GNE***	Global index of neuromuscular balance	Composite index derived from BAR, POC, and TORS values, expressing the overall occlusal stability and neuromuscular equilibrium.	%	Global occlusal stability and neuromuscular efficiency.
***POC SCM***	Percentage Overlapping Coefficient—Sternocleidomastoid	Quantifies the bilateral symmetry of the sternocleidomastoid muscles during clenching, expressed as the percentage overlap between right and left EMG activity.	%	Neuromuscular coordination and functional symmetry of the cervical muscles.
***CERVICAL LOAD***	Cervical Load Index	Quantifies the relative recruitment of the sternocleidomastoid muscles during maximum voluntary clenching (MVC), expressed as the electromyographic contribution of the cervical muscles relative to the overall activity of the mandibular elevator muscles (bilateral masseter and anterior temporalis muscles).	%	Functional cervical involvement and neuromuscular cranio-cervical compensation.

**Table 3:** Teethan Parameters (Chewing Test).

*Index*	*Full Name*	*Definition*	*Unit*	*Represents*
***FREQ***	Masticatory Frequency Index	Quantifies the number of masticatory cycles performed within a defined time interval during the chewing test.	cycles/s (Hz)	Rhythmicity and functional efficiency of masticatory activity.
***IMPACT TA***	Temporalis Impact Index	Represents the overall magnitude of electromyographic activation of the anterior temporalis muscles during mastication, derived from the cumulative EMG amplitude generated throughout the chewing task.	%	Functional workload and activation level of the temporalis muscles during mastication.
***IMPACT MM***	Masseter Impact Index	Represents the overall magnitude of electromyographic activation of the masseter muscles during mastication, derived from the cumulative EMG amplitude generated throughout the chewing task.	%	Functional dominance and muscular workload of the working side during chewing activity.
***IMPACT WL***	Working Side Impact Index	Expresses the relative electromyographic contribution of the muscles on the working side during mastication, calculated from the integrated EMG activity generated throughout chewing cycles.	%	Functional lateralization and neuromuscular efficiency during mastication.
***SMI***	Symmetrical Mastication Index	It compares the neuromuscular coordination in the two tests. The optimal value is 100%, the further we move away from the ideal value, the greater the chewing disorder.	%	Single index which indicates the chewing coordination

The level of statistical significance was set at *p* < 0.05.

**Table 4:** Electromyographic Parameters of the Occluso-Cervical Test in Professional Rally Drivers.

*Parameters*	*Mean ± SD*	*Median [IQR]*	*Min*	*Max*	*Range*	*CI*
***POC TA***	84.6 ± 4.17	86.5 [5.35]	74.0	88.5	83 ≤ (%) ≤ 100	82.62, 86.60
***POC MM***	83.4 ± 7.01	84.6 [5.61]	58.8	90.5	83 ≤ (%) ≤ 100	79.96, 86.94
***BAR***	86.3 ± 4.57	86.9 [6.24]	75.0	91.9	90 ≤ (%) ≤ 100	83.67, 88.85
***TORS***	88.7 ± 3.95	89.8 [2.34]	75.7	92.5	90 ≤ (%) ≤ 100	86.69, 90.67
***IMPACT***	92.3 ± 32.2	94.0 [41.1]	30.4	166	85 ≤ (%) ≤ 115	76.43, 108.23
***ASIM***	−4.26 ± 7.63	−6.42 [9.56]	−13.9	10.9	−10 ≤ (%) ≤ 10	−7.84, 0.12
***GNE***	85.9 ± 3.18	86.0 [4.50]	77.0	91.0	83 ≤ (%) ≤ 100	84.40, 87.38
***POC SCM***	78.3 ± 10.1	80.1 [8.39]	50.5	87.5	83 ≤ (%) ≤ 100	73.36, 83.30
***Cervical Load***	17.5 ± 16.4	13.4 [12.1]	1.77	62.4	0 ≤ (%) ≤ 15	9.52, 25.44

**Table 5:** Static Electromyographic Parameters of the Chewing Test in Professional Rally Drivers.

*Parameters*	*Mean ± SD*	*Median [IQR]*	*Min*	*Max*	*CI*
***FREQ DX***	1.61 ± 0.163	1.56 [0.250]	1.40	1.99	1.53, 1.69
***FREQ SX***	1.72 ± 0.192	1.74 [0.230]	1.41	2.16	1.63, 1.81
***IMPACT TA DX/SX***	120 ± 68.2/ 94 ± 69.4	96.6 [54.8]/ 85.2 [60.4]	20.8/1.78	314/306	85.67, 153.51/ 59.97, 128.97
***IMPACT MM DX/SX***	141 ± 106/ 127 ± 90.9	105 [74.1]/ 108 [70.3]	53.9/37.7	510/441	88.54, 193.74/ 81.76, 172.10
***IMPACT WL DX/SX***	1160 ± 487/ 1114 ± 486	1049 [552]/ 1114 [441]	428/3.54	2214/2008	917.79, 1401.85/ 872.70, 1356.24
***SMI***	51.4 ± 18.7	55.0 [28.5]	18.0	81.0	41.56, 60.44

**Table 6:** Dynamic Electromyographic Parameters Recorded During the Competition.

*Parameters*	*Mean ± SD*	*Median [IQR]*	*Min*	*Max*	*CI*
***A-MWI***	5.06 ± 3.58	3.53 [3.08]	1.45	14.5	3.07, 7.05
***A-MTI***	19.4 ± 9.83	18.9 [8.84]	9.14	53.1	14.47, 24.41
***A-MPI***	14.6 ± 7.43	14.1 [7.15]	6.62	38.9	11.17, 18.13
***A-BWI***	2.70 ± 4.05	1.39 [1.51]	0	14.8	0.71, 4.69
***A-BTI***	2.09 ± 2.64	1.40 [1.90]	0	11.7	0.60, 3.58
***A-BPI***	6.57 ± 18.5	1.53 [2.39]	0	82.0	−2.88, 16.02
***Clenching Episodes***	37.9 ± 46.2	21.0 [24.5]	0	149.0	14.96, 60.94
***Grinding Episodes***	6.53 ± 12.1	3.00 [7.50]	0	53.0	0.52, 12.54

**Table 7:** Main Statistical Analyses of Neuromuscular and Stomatognathic Parameters.

*Analysis*	*Statistical Method*	*Result*	*Significance*
***Clenching Episodes vs. Grinding Episodes***	Wilcoxon signed-rank	The number of clenching episodes was significantly greater than that of grinding episodes	*p* < 0.001
***Cervical Load vs. Clenching Episodes***	Spearman	Moderate positive correlation	*p* = 0.011
***Cervical Load vs. A-MTI***	Spearman	Positive but non-significant correlation	*p* = 0.206
***SMI vs. A-BPI***	Spearman	No significant correlation	*p* = 0.954

## Data Availability

The raw data supporting the conclusions of this article will be made available by the authors on request.
